# Overview of the Treatment Goal, the Method of Evaluating Disease Activity/Physical Function, Activities of Daily Living, and Traditional Care for Systemic or Articular Juvenile Idiopathic Arthritis in Japan

**DOI:** 10.3390/children11080952

**Published:** 2024-08-06

**Authors:** Masaaki Mori

**Affiliations:** 1 Department of Lifetime Clinical Immunology, Graduate School of Medical and Dental Sciences, Tokyo Medical and Dental University, 1-5-45 Yushima, Bunkyo-ku, Tokyo 113-8519, Japan; mori.phv@tmd.ac.jp; Tel.: +81-03-5803-4876; Fax: +81-03-5803-4694; 2 Division of Rheumatology and Allergology, Department of Internal Medicine, St. Marianna University School of Medicine, Kawasaki 216-8511, Japan

**Keywords:** juvenile idiopathic arthritis, treatment goal, disease activity, physical function, traditional care

## Abstract

Juvenile idiopathic arthritis (JIA) is a chronic arthritis of unknown cause that develops in patients younger than 16 years of age and persists for at least 6 weeks. It is an important cause of short- and long-term physical and mental impairments in children. The goal of treatment for JIA is remission. A T2T (treatment-to-target) has been proposed and practiced as a means of achieving remission. The method of evaluating the disease activity of JIA depends on the disease type. For systemic JIA, disease activity is determined by comprehensively considering joint findings, systemic inflammatory findings, changes in inflammatory and synovitis markers, imaging findings, and other factors. For articular JIA other than systemic JIA, the Juvenile Arthritis Disease Activity Score (JADAS-27) is used to evaluate disease activity. The CHAQ (Childhood Health Assessment Questionnaire) and the Japanese version of the modified Rankin Scale (mRS) are mainly used to assess the physical function and ADL. The CHAQ is a global standard assessment method with the advantage that it can be transitioned to the HAQ used in adults, making it useful for international comparisons. The mRS is used to classify the severity of JIA as a chronic disease, and is an indispensable evaluation method in the specific disease procedure in Japan. It is necessary to have pediatric-specific knowledge of growth and development and routine childhood immunizations and to consider transition support tailored to the patient’s situation. Ultimately, the goal is to foster the patient’s independence and to provide an uninterrupted follow-up in the adult care department. Continuous follow-up will be provided during the schooling (and later, employment) period, and the relationship with the patient will be tailored to their developmental stage. It is also important to understand and communicate the importance of contraception and the drugs that cannot be used during pregnancy.

## 1. What Kind of Disease Is Juvenile Idiopathic Arthritis (JIA)?

### 1.1. Concepts and Classification

JIA is a chronic arthritis of unknown cause that develops in patients younger than 16 years of age and persists for at least 6 weeks. According to the ILAR classification criteria revised in 2001, the disease is classified into seven types: (i) systemic, (ii) oligoarthritis (persistent or progressive), (iii) rheumatoid factor (RF)-negative polyarthritis, (iv) RF-positive polyarthritis, (v) psoriatic arthritis, (vi) enthesitis-related arthritis, and (vii) unclassified arthritis [1,2]. JIA is a chronic disease that fulfills the following four conditions: (1) the mechanism of pathogenesis is unknown, (2) treatment is not fully established, but is becoming more established, (3) the disease is rare, and (4) the disease requires long-term medical treatment. When initial treatment is delayed, treatment is inadequate, or the disease is refractory, complications of the disease (joint destruction and uveitis) and side effects of medications (mainly corticosteroids) can reduce the quality of life of the affected child throughout their life. Recently, however, diagnostic and therapeutic techniques and methods have advanced remarkably with the development of diagnostic imaging equipment such as joint ultrasonography and MRI, as well as the field of immunology, and good prognosis can now be expected. Finally, I believe that we should aim for Dreams Come True Remission in order to help JIA patients realize their future dreams [3].

The pathogenesis of JIA can be largely categorized into two types: the systemic type (corresponding to (i) above), which is mainly of autoinflammatory pathology, and the joint type (corresponding to (ii) to (iv) above), which is mainly of autoimmune pathology. Each type of JIA has its own characteristics, such as age of onset, gender, associated symptoms, arthritis pattern, treatment methods, complications, and prognosis. Therefore, it is necessary to be aware of these characteristics when treating patients with JIA.

When comparing these seven JIA types with rheumatoid arthritis (RA), it can be seen that oligoarthritis and RF-negative polyarthritis have different characteristics from RA, while the RF-positive polyarthritis type more closely resembles RA. In addition, the systemic type is also called adult-onset Still’s disease when it occurs in adulthood and might be considered to be the same condition with the only difference being the age of onset.

### 1.2. Epidemiology

The overall prevalence of JIA in Japan is 10 to 15 per 100,000 children. The frequency of each type of JIA was determined in a 2008 survey of recipients of the Medical Expense Subsidy System for Chronic Diseases of Children. The systemic type, oligoarthritis, RF-negative polyarthritis, and RF-positive polyarthritis account for more than 90% of the cases. On the other hand, a questionnaire survey of pediatric rheumatologists in 2016 revealed that enthesitis-related arthritis and psoriatic arthritis, which were previously considered rare, also exist to some extent. There are also characteristic differences in sex and age of onset depending on the type of disease. The peak age of onset and sex difference by major disease type is 1–5 years for systemic type (no sex difference), 5.8 years for oligoarthritis (sex ratio = 1:2.5), 7.0 years for RF-negative polyarthritis (sex ratio = 1:2.2), and 9.9 years for RF-positive polyarthritis (sex ratio = 1:8.0) in Japan [4]. In other words, oligoarthritis and polyarthritis are more common in female children.

### 1.3. Pathophysiology

The pathophysiology of systemic JIA is completely different from that of joint-type JIA. Systemic JIA is mainly an “autoinflammatory disease” with a background of abnormal innate immunity and repeated systemic inflammation. This involves excessive production of systemic proinflammatory cytokines. Articular-type JIA is an “autoimmune disease” against a background of abnormal acquired immunity to cartilage-derived autoantigens.

At the site of arthritis, there is persistent inflammation of the tissue called the synovium, and cartilage and bone destruction due to infiltration of inflammatory cells and proliferation of the synovium is observed [5]. This involves the production of inflammatory cytokines at the site of arthritis.

Genetic factors have been suggested based on the racial differences in the incidence of JIA by disease type, the high incidence in females, the increased incidence among siblings, and the association with human leukocyte antigen (HLA) and genes other than HLA. For instance, HLA A2, DR, and DRB1 are reported to be associated with JIA, and PTPN22 and PTPN2 gene mutations are reported to be associated with JIA for genes other than HLA. However, children with these HLA and non-HLA gene mutations do not always develop JIA. Acquired factors after birth, i.e., environmental factors, are considered to play a greater role. Environmental factors such as breastfeeding, exposure to antimicrobial agents, infectious diseases, and maternal smoking have been implicated, but these are not known for certain [6].

## 2. What Is the Treatment Goal of JIA?

### 2.1. What Is the Treatment Goal?

The goal of treatment for JIA is remission. Remission is a stable and settled state in which symptoms and abnormalities on tests have disappeared. No established treatment for JIA leads to a “cure”, which is a state of complete recovery, and the basic goal is to maintain remission with continued treatment. Therefore, the term “cure” is generally not used [5]. Maintaining remission suppresses the progression of joint destruction, improves activities of daily living (ADL), maximizes quality of life, and improves long-term prognosis [7].

Remission criteria are variable depending on the type of JIA considered, given the great heterogeneity of its different clinical forms. Remission is determined by assessing disease activity. The Wallace criteria are used for systemic and joint-type JIA, and the JADAS-27 is also often used for joint-type JIA.

In order to prevent functional disability, it is necessary to achieve remission early by strictly controlling disease activity, i.e., tight control. In rheumatoid arthritis (RA), a T2T (treatment-to-target) strategy has been proposed and practiced as an approach [7].

### 2.2. What Is T2T?

T2T refers to a “treatment-to-target” strategy. T2T strategies with an awareness of target values have led to a better prognosis [6]. This concept was proposed for RA in 2010, and the direction of this concept was also proposed for JIA in 2018 [8].

T2T consists of “Basic Principles” (Table 1) and “Recommendations” (Table 2). Specifically, the first goal of treatment is clinical remission, and in patients with long-term disease, the goal is to achieve low disease activity. Therefore, we regularly and appropriately use indices to determine the effectiveness of treatment. Once the goal is achieved, the next goal is to maintain the condition, thereby keeping the QOL in a good state. Our basic principle is that the patient and physician work together to decide on a treatment plan to achieve these clear goals throughout treatment.

## 3. What Methods Are Available for Assessing Disease Activity Levels of JIA?

### Indices of Remission and Assessment of Disease Activity by Disease Type

Different scores are used to assess the disease activity of JIA depending on the type of disease.

The remission criteria of Wallace et al. in 2011 (ACR remission criteria) are often used as an index of remission, regardless of the disease type. Under these criteria, a patient is considered to have inactive disease (ID) if all of the following conditions are met: (i) no active arthritis, (ii) no fever, rash, serositis, splenomegaly, or lymphadenopathy associated with JIA, (iii) no uveitis, (iv) normal ESR or CRP range, and (v) best overall evaluation by the physician. If all of the above conditions are met, plus (vi) morning stiffness of 15 min or less, the patient is considered to have clinical inactive disease (CID). The status of ID lasting more than 6 months while receiving treatment is defined as clinical remission on medication (CRM), and the status of ID lasting more than 12 months without any treatment is defined as clinical remission off medication (CR) [9,10].

On the other hand, since there is no consensus index of disease activity in systemic JIA, disease activity is evaluated by comprehensively considering the findings of joints, systemic inflammation, inflammatory markers, synovitis markers, and imaging findings.

The JADAS-27 is mainly used to evaluate the disease activity of JIA other than the systemic type, excluding psoriatic arthritis and enthesitis-related arthritis, and is also adopted in the designated intractable disease severity classification of JIA [11]. The JADAS-27 cut-off values are ≤1.0 for remission, ≤2.0 for low activity, 2.1 to 4.2 for moderate activity, and >4.2 for high activity.

The following is a difference from the evaluation of rheumatoid arthritis (RA). The 28 joints used for evaluation in the Disease Activity Score 28 joints (DAS28), the Simplified Disease Activity Index (SDAI), and the Clinical Disease Activity Index (CDAI), which are commonly used for rheumatoid arthritis (RA), do not include cervical joints (approximately 5% to 20%) or ankle joints (approximately 25% to 60%), which are frequently found in JIA. Therefore, it is generally not recommended to use these evaluation methods for JIA patients.

The JADAS-27 can be automatically calculated in the “Society Activities—Clinical Support Tools” on the website of the Pediatric Rheumatology Association in Japan [12].

## 4. What Methods Are Used to Assess Physical Function and ADLs in JIA?

### CHAQ and mRS

In JIA clinical practice, the CHAQ is mainly used as an objective functional assessment method for physical dysfunction. This is a functional assessment index that can be used for patients 19 years of age or younger, is simple enough to be completed in less than 10 min, and has properties that allow it to be transitioned to the HAQ after the age of 20.

The actual CHAQ consists of 30 questions about eight functional categories (grooming, waking, eating, walking, hygiene, reaching, grasping, and activity). The answers to the questions are scored, and the highest score in a domain is the score for that domain, and the average is calculated to give the CHAQ score. A score of 0 is given if the patient can perform activities of daily living without any difficulty at all. The items that are generally considered too difficult at the child’s age are excluded from the evaluation by checking “Not Applicable”. The parent or guardian may also fill in the form depending on the age of the child.

The Japanese version of the CHAQ 2019 is available for download from the website of the Pediatric Rheumatology Association in Japan under “Society Activities—Clinical Support Tools” [13].

On the other hand, the Japanese version of the modified Rankin Scale (mRS) is used in the JIA classification of the severity of designated intractable diseases [14]. This evaluation method has been adopted in the classification of the severity of designated intractable diseases for many diseases to compare them with other diseases. The table shows a comparison with Steinbrocker’s criteria for the classification of functional disability, which has been commonly used in rheumatoid arthritis [15] (Table 3 and Table 4).

## 5. Traditional Care

### 5.1. Pediatric Knowledge

In addition to patients with JIA who are growing and developing, there are a variety of other chronic pediatric diseases that require transitional care (metabolic endocrine, cardiovascular, renal, neurological, hematologic, chronic diseases diagnosed in the perinatal period, etc.). Still, they all have one thing in common: the normal growth and development of the child. The developmental period is divided into the neonatal period (from birth to 4 weeks), infancy (from birth to 1 year), early childhood (until the start of elementary school), school age (childhood: the period during which children attend elementary school), and adolescence (from junior high school to adolescence or until height growth stops). In general, physical development (growth) is followed up at the infant health examination until early childhood and at the school health examination after that.

Since prolonged inflammation and long-term use of corticosteroids may cause growth retardation in pediatric patients with rheumatic diseases, it is necessary to check their body size against the growth curve. Regarding mental development, there are several developmental tests. However, not all children take them, so we have to ask questions such as “What is your grade level, and are you in a regular class?”, “Is he/she living in the same school environment as other children?”, and “Do they have any good friends?” to estimate their development level. Patients tend to spend their growing years under the deep love and protection of their parents and may be more immature and dependent than normal children of the same age.

In addition, routine vaccinations are given to children from infancy to school age. Still, it is possible that some children may not have been vaccinated depending on their medical conditions at that time or the drugs used. If so, it is important to check how they are following up and what they plan to do in the future.

### 5.2. What Is “Transitional Care?”

The term “transitional care” has become a hot topic in recent years. This is because, due to advances in medical care, many patients with childhood-onset chronic diseases are now entering adulthood, and both pediatric and adult diseases are coexisting. Pediatric rheumatic diseases including JIA also fall under this category [16]. A problem that arises is the difference between pediatric care and adult care. Pediatric care differs significantly from adult care, especially in the physical and mental changes accompanying growth and development, the need for multiple routine immunizations, and the presence of parents and caregivers to interact with patients. There have been cases where transition was not possible due to these issues, and a support system with medical staff knowledgeable in transitional medicine needed to be enhanced.

### 5.3. When Is the Appropriate Transition Period?

A transition model plan has not yet been established in Japan. In the United States, the “Six Core Elements of Health Care Transition 3.0” is listed as a methodology, in which a transition plan is presented to the patient at around 12 to 14 years of age. Since there are racial and cultural differences, it is necessary to consider each case in Japan while referring to the methodology described above [17]. At this stage, the web-based “Transition support and independence support information sharing website” [18] reports a core guide that includes an introduction to the tools, which may be helpful.

### 5.4. Transitional Goals

**Figure 1:**
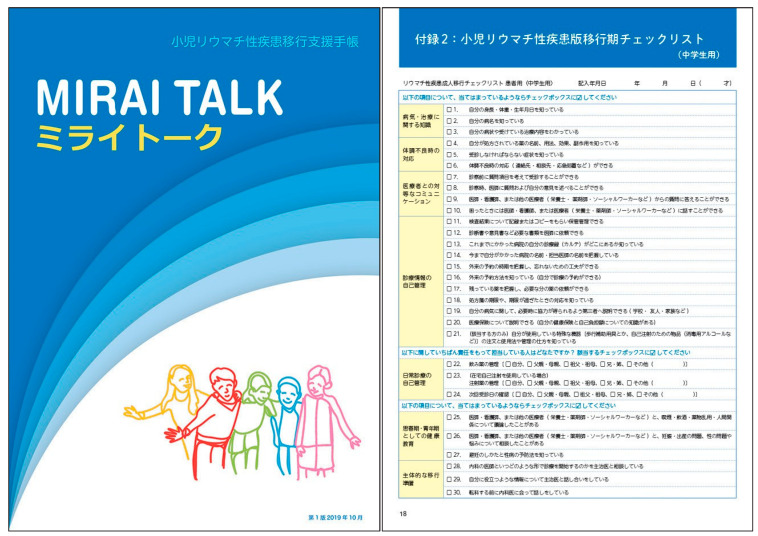
MIRAI TALK, a handbook to support the transition of pediatric rheumatic diseases. Adapted from Reference [5].

Each patient needs to be followed up continuously by the adult care department which needs to seek ways to relate to the patient. There is a handbook called “MIRAI TALK” (Figure 1) which helps providers keep track of the patient’s long-term progress and foster their independence. It is advisable to check its contents since it can be downloaded from the website of the Japan College of Rheumatology [19]. We hope that many patients will be able to explain the name of the disease, age of onset/symptoms, course, treatment details, and complications, and be able to manage medications, including self-injection and oral medication thanks to the provider guidance shared in this handbook [5].

## 6. Health Education

### 6.1. What Knowledge Should the Medical Staff Have and How Should They Talk to the Patient?

The situation of patients with chronic diseases differs greatly depending on the timing of disease onset, duration of disease, disease status, and complications. Pediatric knowledge may be necessary for growing and developing pediatric patients, as described above. In addition, school attendance, employment, pregnancy (including contraception), and childbirth should also be followed up.

### 6.2. School Attendance and Job Placement

Childhood-onset cases may not be able to attend school due to physical complications, drug effects, or prolonged treatment. When enrolling in or transferring to a new school, it is necessary to confirm the possibility of needing to change JIA physicians and the availability of adequate treatment at the new school in advance. It is desirable to be able to provide job placement information, as there may be support available, such as job placement assistance, depending on the situation.

### 6.3. Pregnancy (Including Contraception) and Childbirth

Some medicines may not be used during pregnancy. The main drugs used in rheumatic diseases include methotrexate (MTX; Rheumatrex^®^), mycophenolate mofetil (CellCept^®^), leflunomide (Arava^®^), and Mizoribine (Bredinin^®^), with MTX being the most commonly used drug in JIA. Nonsteroidal anti-inflammatory drugs (NSAIDs) are also contraindicated in late pregnancy (after 28 weeks) because they may constrict the ductus arteriosus necessary for fetal blood flow. Janus kinase (JAK) inhibitors are also considered an effective future treatment, but their safety during pregnancy has not been confirmed. It is the responsibility of the health care providers to make sure that the patients understand the importance of the contraindications. Recently, “preconception care”, in which health care is emphasized for future pregnancies regardless of the presence or absence of underlying diseases, has been attracting attention and is expected to be enhanced.

Knowledge of contraception is also necessary when the above drugs are used, when there is no desire to have a baby when the disease activity is high, or when a controlled pregnancy and delivery are required. Intrauterine systems and oral contraceptives are the most reliable methods of birth control, followed by the use of condoms, which are also effective in preventing sexually transmitted diseases. Note, however, that oral contraceptives are contraindicated if there is a risk of thrombosis, such as antiphospholipid antibody syndrome [16]. In Japan, there is a lack of specific instruction on contraceptive methods in sex education. It is a good idea during this transitional period to make sure that the child knows about each contraceptive method and why it is used.

## Figures and Tables

**Table 1:** Basic principles of T2T.

A	Treatment goals and strategies should be agreed upon by the parents/patient and the pediatric rheumatology team.
B	JIA is a heterogeneous group of diseases and requires a clear therapeutic approach.
C	The treatment goals for JIA patients are to control symptoms, prevent structural damage, avoid complications and side effects of medications, and optimize physical function, growth and development, quality of life, and participation in social activities.
D	To achieve these goals, it is essential to reduce inflammation.
E	Prolonged use of systemic corticosteroids to maintain goals should be avoided.
F	To achieve these goals, regular assessment of disease activity and modification of treatment accordingly is important.

**Table 2:** Recommendations for T2T.

1	The treatment goal for JIA is to achieve clinical remission, i.e., to achieve a state in which there are no clinical signs or symptoms of disease activity, including extra-articular symptoms.
2	Reducing disease activity to a low level may be an immediate treatment goal, especially in patients with prolonged disease.
3	The setting of treatment goals and the selection of treatment methods should be based on the individual characteristics of each patient and agreed upon with the parents/patient.
4	Assessment of disease activity should be performed and documented regularly using composite indices.
5	The frequency of evaluation depends on the type of JIA, the degree of disease activity, and the presence of extra-articular symptoms. Patients with systemic JIA and high disease activity should be evaluated weekly, patients with moderate to high disease activity every 1 to 3 months, and less frequently in patients who are in clinical remission.
6	All patients should achieve a 50% or greater improvement in disease activity within 3 months and achieve the established treatment goal within 6 months. In patients with systemic JIA and high disease activity, the goal should be to achieve resolution of fever within 1 week.
7	Treatment must be adjusted and reviewed until goals are achieved.
8	Once treatment goals are achieved, they must be maintained. Therefore, continuous monitoring should be carried out.

**Table 3:** The Japanese version of the modified Rankin Scale (mRS) decision criteria.

Modified Rankin Scale		Points of Reference
0	There are no symptoms at all.	There are no subjective symptoms or objective signs of the condition.
1	Symptomatic but no obvious disability: Able to perform daily tasks and activities.	Subjective symptoms and objective signs are present, but there are no restrictions on work or activities that the patient had been partaking in before the onset of the disease.
2	Mild disability: Not able to perform all the activities they were able to perform before the onset of the disease, but able to perform their daily activities without assistance.	The patient is independent in daily life, although there are some limitations in the work and activities that they had been engaged in prior to the onset of the disease.
3	Moderate disability: Requires some assistance but can walk without assistance.	The patient needs assistance with shopping and going on public transportation, but does not need assistance with usual walking, eating, grooming, toileting, and other activities.
4	Moderate to severe disability: Walking and physical demands require assistance.	The patient requires assistance with usual walking, eating, grooming, toileting, etc., but does not require continuous nursing care.
5	Severe disability: Bedridden, incontinent, requiring constant care and monitoring.	Always in need of assistance from someone.
6	Death	

**Table 4:** Steinbrocker’s criteria for classification of functional disability (reference).

Class I	Physical functions are complete, and the patient can perform all normal tasks without any disability.
Class II	The patient may have pain in one or more joints during movement or may have limited movement but be able to manage normal activities.
Class III	The patient can perform normal work and personal tasks with little or no ability to do so.
Class IV	The patient is bedridden or uses a wheelchair, with little or no ability to perform daily personal activities.
